# Effects of Sodium Hypochlorite Rinsing on Tilapia Storage: An Investigation Based on Muscle Quality and Tissue Protease Activity

**DOI:** 10.3390/foods14162868

**Published:** 2025-08-19

**Authors:** Zirui Fu, Shuxian Hao, Huan Xiang, Chunsheng Li, Jianwei Cen, Ya Wei, Shengjun Chen, Yongqiang Zhao, Xiao Hu, Yuhong Yan, Hui Huang, Jun Li

**Affiliations:** 1 Key Laboratory of Aquatic Product Processing, Ministry of Agriculture and Rural Affairs, South China Sea Fisheries Research Institute, Chinese Academy of Fishery Sciences, No. 231, Xingang West Road, Haizhu District, Guangzhou 510300, China; fzrui2333@163.com (Z.F.); susanhao2001@163.com (S.H.); skyxianghuan@163.com (H.X.); lichunsheng@scsfri.ac.cn (C.L.); genvex@163.com (J.C.); weiya@scsfri.ac.cn (Y.W.); chenshengjun@scsfri.ac.cn (S.C.); zhaoyq@scsfri.ac.cn (Y.Z.); hnhuxiao@163.com (X.H.); wluck6@126.com (Y.Y.); 2 College of Food Science and Technology, Shanghai Ocean University, Shanghai 201306, China; 3 Key Laboratory of Efficient Utilization and Processing of Marine Fishery Resources of Hainan Province, Sanya Tropical Fisheries Research Institute, Sanya 572000, China; 4 Guangdong Provincial Key Laboratory of Lingnan Specialty Food Science and Technology, Key Laboratory of Green Processing and Intelligent Manufacturing of Lingnan Specialty Food, Ministry of Agriculture and Rural, College of Light Industry and Food, Zhongkai University of Agriculture and Engineering, Guangzhou 510225, China

**Keywords:** tilapia storage, endogenous enzyme activity, hypochlorite rinsing, bacterial reduction, quality characteristics, muscle quality

## Abstract

Sodium hypochlorite solution has a good bacterial reduction effect. Thus, in order to understand the effect of sodium hypochlorite on tilapia storage, in this study, tilapia fillets were treated with sodium hypochlorite and Proclin 300 rinsing, the activity of histatinase protease was measured, and the muscle quality was assessed by the texture, colour, pH, myofibrillar fibril fragmentation index (MFI) and its structural changes. The results showed that sodium hypochlorite rinsing could significantly reduce the activities of histatinase enzymes B, L and D. Meanwhile, the sodium hypochlorite-treated group showed less degradation of myosin heavy chain, pro-myosin and myosin light chain, less degradation of texture and colour, better integrity in the fish myocytes, and a lower myofibrillar fragmentation index (MFI). In conclusion, sodium hypochlorite improved the storage quality of tilapia in several ways. Firstly, it inhibited protease activity, thereby maintaining the structural integrity of fish muscle fibres. Secondly, it reduced the rate of deterioration during storage.

## 1. Introduction

The tilapia (Oreochromis mossambicus) has emerged as a pivotal economic species for freshwater aquaculture in southern China, delivering substantial benefits to practitioners [1]. In recent times, researchers have begun to direct their attention towards the impact of endogenously produced enzymes on the deterioration of texture in aquatic products [2]. Chilling is a prevalent storage method for tilapia prior to processing. Ice storage is a common method used for storing tilapia prior to processing. However, during storage, microbial spoilage and endogenous enzymes catalyse myofibrillar protein degradation, leading to a decline in fish meat quality. This is primarily manifested by changes in the muscle protein structure, degradation, aggregation, and denaturation caused by tissue proteases, resulting in muscle softening and impairing its economic value [3]. Currently, the primary method for preserving fish fillets is freezing at −18 °C. However, freezing can lead to ice crystal growth, which negatively impacts the quality of aquatic products [4], and may also damage the functional properties of myofibrillar proteins [5]. Therefore, how to reduce muscle degradation caused by endogenous proteases under low-temperature conditions has become a key focus of current research.

As Fernandes Lemos Junior et al. [6] demonstrate, sodium hypochlorite solution has been shown to have a significant bactericidal effect. As demonstrated in the relevant literature, this agent, which is commonly used in the processing of tilapia, has a strong oxidising ability. Previous studies have shown that protein oxidation can cause changes in the quality of aquatic products, and ice crystal growth can destroy MP structure. This can, in turn, exacerbate the oxidation state of the MP, leading to disruption to sulfhydryl functional sites, MP self-association, the formation of carbonyl groups, and conformational changes within the protein. The process of ice crystal formation has been demonstrated to induce disruptions in the MP structure, thereby intensifying MP oxidation. This, in turn, has been shown to result in a series of subsequent reactions, including the destruction of sulfhydryl groups, the aggregation of MP, the formation of carbonyl groups, and changes in conformation [7]. Concurrently, research by Gao et al. [8] determined that variations in freezing temperatures and species of fish are a possible cause of differences in the oxidation and denaturation of proteins during storage, and consequently alterations in product quality. Additionally, previous research has confirmed [9] that the oxidation of endogenous enzymes leads to a reduction in their activity, thereby decreasing the degradation of MP and ultimately affecting the preservation of fish meat. Bu et al. [10] found that as the concentration of the oxidant H_2_O_2_ increases, the activity of endogenous enzymes significantly decreases, thereby slowing down the degradation of myofibrillar proteins. The preceding study of our group determined that histatinase B and L are the pivotal target enzymes influencing the textural alterations of tilapia fillets during the freezing process. Histatinase D levels are elevated in tilapia; thus, histatinase B, L, and D were selected as endogenous enzyme indicators in this study.

The aim of the present study was to conduct a thorough investigation of the characteristics of the enzymes present in fish meat during the cold storage process, with a particular focus on the effects of sodium hypochlorite treatment. The investigation will concentrate on the alterations in histatinase protease activities B, L, and D in fish meat after oxidative sterilisation reduction treatment. Furthermore, the study will analyse their impact on the ultrastructure of myofibrils and the mechanism of their action on protein degradation. The selection of treatment conditions was based on the findings of the preceding study, which demonstrated an 80% bacterial reduction rate. The experimental group was subjected to oxidative sterilisation using 200 mg/L sodium hypochlorite for a duration of 10 min, while the control group underwent sterilisation with Proclin 300 for an equivalent duration. This approach ensured that the bacterial reduction rate in both groups was comparable [11]. In the present study, the myofibril proteostructures, colours, MFI, SDS-PAGE electrophoresis, activity of histatinase B, L and D, and ultrastructure of the myofibrils of fish meat during 4 °C refrigeration were observed. The effect of sodium hypochlorite on protein structure and muscle tissue was then analysed, with the aim of elucidating the role of sodium hypochlorite oxidation on the changes in the quality of fillets of tilapia.

## 2. Materials and Methods

### 2.1. Materials

The fish used in this study was fresh tilapia from CR Vanguard (Guangzhou, China); Z-pHe-Arg-AMC,Z-Arg-Arg-AMC was from Sigma-Aldrich (St. Louis, MO, USA); Histatinase B, L and D were from Nearshore Technology (Shanghai, China); and Proclin 300 preservative and bovine haemoglobin were from Solarbio (Beijing, China). The other reagents are commonly used in the laboratory.

### 2.2. Sample Processing

Fresh tilapia (800 ± 50 g) were subjected to a stun and slaughter process, after which the skin was removed and the back muscles removed. Subsequent to this, the fish were divided into fillets, with each fillet exhibiting dimensions of 10.0 cm × 4.0 cm × 0.5 cm, and subsequently cleaned. The fish meat was divided into two groups and soaked in a solution with a ratio of 1:5. The experimental group was soaked in a 200 mg/L sodium hypochlorite solution for 10 min, while the control group was soaked in a solution containing Proclin 300 for 10 min, so that the sterilisation rate of the control group was consistent with that of the sodium hypochlorite solution. The processed fish fillets were weighed, packaged separately, and stored in a refrigerator (−4 °C). For analysis, the samples were collected at fixed storage times (0, 1, 2, 3, 4, 5, and 6 days). Three fish fillets were selected from each treatment for soaking and frozen storage. Each fish fillet sample was measured three times in parallel and the average value was taken.

### 2.3. Extraction of Myofibrillar Protein (MP)

The present study will follow the method described by Pazos et al. [12], with appropriate modifications. Following the mincing of the fish meat, 2 g of sample from different treatments were accurately weighed and placed in 50 mL centrifuge tubes. The following steps were taken in order to complete the procedure: firstly, 10 mL of Tris-HCl buffer (containing 10 mmol/L Tris-HCl and 1 mmol/L PMSF, pH 7.2, 4 °C) was added. Then, the homogenate was created by means of a homogeniser at a speed of 10,000× *g* for 10 min, under ice bath conditions. The homogenate was then subjected to centrifugation at 10,000× *g* for 20 min (4 °C). Following the process of centrifugation, the upper layer was discarded. Thereafter, 20 mL of a salt solution (comprising 0.6 mol/L NaCl, 10 mmol/L Tris-HCl buffer, 1 mmol/L PMSF, pH 7.2, 4 °C) was added to the precipitate. The precipitate was then homogenised and thoroughly mixed. The mixture was then incubated on ice for 30 min. Thereafter, it was subjected to centrifugation at 10,000× *g* for 20 min (4 °C). The resultant upper layer, which is the myofibrillar protein solution, was then collected. The protein concentration was determined using the BCA method, and the resultant solution was stored at −80 °C.

### 2.4. Extraction and Activity Determination of Tissue Proteases B and L

In accordance with the methodology outlined by Fabrizi et al. [13], a quantity of 5 g of fish meat was taken and combined with 20 mL of pre-cooled Tris-HCl buffer solution (20 mmol/L, pH 7.5). The mixture was then homogenised and subjected to a centrifugation process for a duration of 20 min at an angular velocity of 10,000× *g* at a temperature of 4 °C. The enzyme solution of histatinase B and L was obtained by subjecting the floating fat to filtration through gauze. Concurrently, the buffers for histatinase B and L were prepared in accordance with the method described by Zhang et al. [14]. The following is the protocol for preparing a buffer solution of histatinase B: A mixed solution consisting of 352 mmol/L KH_2_PO_4_, 48 mmol/L Na_2_HPO_4_, and 4 mmol/L Na_2_EDTA was prepared, with the solution adjusted to a pH of 6.0. Prior to use, 8 mmol/L L-cysteine was added, followed by thorough mixing to ensure uniform distribution. The following is a list of the components required for the preparation of a buffer solution for histatinase L: A mixed solution should be prepared, comprising 340 mmol/L NaAc, 60 mmol/L HAc, and 4 mmol/L Na_2_EDTA, with the pH maintained at 5.5. Prior to use, the addition of 8 mmol/L DTT is required, followed by thorough mixing to ensure uniform distribution. The following solution is to be employed in order to bring the matter to a conclusion: The solution was composed of 100 mmol/L ClCH_2_COONa, 30 mmol/L NaAc, and 70 mmol/L HAc, with a pH of 4.3.

Protease activity was determined according to the method of Zeng et al. [15]. In order to execute the experiment, 0.5 mL of crude enzyme solution was added to 0.25 mL of reaction buffer. The mixture was then preheated at 37 °C for 10 min. Subsequently, 0.5 mL of 20 μmol/L substrate, which had been preheated for 10 min, was added (the specific fluorescent substrates for histatinase B and L are Z-Arg-Arg-AMC and Z-pHe-Arg-AMC, respectively). The mixture was then thoroughly mixed and incubated at 37 °C for 30 min. The stop solution was added to the sample, after which the mixture was subjected to centrifugal force at 10,000× *g* and 4 °C for a period of 15 min. The resultant clear layer was then collected. The AMC release was measured using an F-7000 fluorescence spectrophotometer (Hitachi, Japan) under the following conditions: excitation wavelength (λex) 340 nm and emission wavelength (λem) 440 nm.

### 2.5. Extraction and Activity Assay of Tissue Protease D

As outlined by Liburdi et al. [16], the extraction of histatinase D is achieved through the following steps. Firstly, 15 g of fish meat is taken. Then, 150 mL of pre-cooled acetone (−40 °C) is added. The mixture is then homogenised and the homogenate filtered through a Büchner funnel. The filtrate is then air-dried under a fume hood for 12 h to make acetone powder. The addition of 20 millilitres of a 2% KCl solution to the acetone powder is required, followed by homogenisation and centrifugation for 15 min at 10,000 revolutions per minute at 4 degrees Celsius. This process ultimately yields a liquid known as histatinase D, as evidenced by Yan et al. [11], albeit with minor adjustments to the methodology. In this experiment, 5% acid-denatured bovine haemoglobin was utilised as the substrate. Subsequently, 0.5 mL of crude histatinase D enzyme solution was added to 0.5 mL of acid-denatured haemoglobin and 1.5 mL of McKelvin’s buffer (pH 3.0). The next stage of the experiment involved subjecting the mixture to thermal stimulation at 37 °C for a duration of one hour. This was followed by the termination of the reaction by the introduction of 2.5 mL of TCA (5%). Thereafter, the reaction mixture was left at room temperature for a period of one hour. Subsequently, the mixture was submitted to a centrifugal process at a speed of 7000 r/min and a temperature of 25 °C for a period of 20 min. The UV absorption of the filtered sample was ascertained at a 280 nm wavelength, using a Multiskan^FC^ enzyme marker (Guangzhou, China).

### 2.6. Determination of Texture

In this study, the methodological approach of Zhuang et al. [17] was referenced and modified in a suitable manner. The QTS-25 texture tester (Guangzhou, China) was utilised to ascertain the texture of fish fillets under various attenuation treatments. In the present study, three fish fillets were obtained from each group. Five positions along the diagonal of the fillets on the dorsal side were then selected for the test. The test mode employed was texture multi-faceted profiling. The probe used was a flat-bottomed TA44 cylindrical probe. In the experimental setting, the test speed was established at 1.0 mm/s, and the number of cycles was set to 2. Furthermore, it was observed that the trigger point load registered at 5.0 g, whilst the downward pressure distance was set at 5.0 mm.

### 2.7. Determination of Colour and Lustre

It is evident that the methodology employed by Nie et al. [18] was referenced, with suitable modifications being implemented. The L*, a*, and b* values of fish fillets subjected to various reduction treatments were determined at ambient temperature by employing a CM-200S colourimeter (Guangzhou, China).

### 2.8. Determination of pH

The method under discussion is that set out in GB 5009.237-2016, entitled “National Standard for Food Safety: Determination of pH Value of Food”, with appropriate modifications [19]. Five grams of fish meat should be added to 50 millilitres of distilled water, with the mixture then being homogenised. The resultant mixture should then be left to stand for five minutes before being determined by means of a pH metre.

### 2.9. Determination of Myofibrillar Fibril Fragmentation Index (MFI)

The methodology outlined by Prill et al. [20] was followed, with suitable modifications being made. The MP solution was then diluted to a concentration of 0.5 mg/mL using MFI buffer (Guangzhou, China) (8.8 mmol/L KH_2_PO_4_, 11.2 mmol/L K_2_HPO_4_, 1 mmol/L EGTA, 100 mmol/L KCl, 1 mmol/L MgCl_2_. The assay was performed using a Multiskan FC enzyme-linked immunosorbent assay reader (Guangzhou, China) with a measurement of the optical density at 540 nm. The MFI content was calculated as the OD value multiplied by 200.

### 2.10. SDS-PAGE Analysis

This study adapted the approach outlined by Pan et al. [21], with modifications to the MP amalgamation process to ensure its compatibility with the SDS-PAGE loading buffer (5×). The volumetric ratio of the MP to the SDS-PAGE loading buffer was set at 3:2 (*v*/*v*). The substance was then exposed to heat in a water tank during the course of five minutes. The loading volume was set at 10 μg, and electrophoresis was conducted using a 12% SDS-PAGE separator gel (Shanghai, China), a 5% concentrated gel, an electrophoresis voltage of 120 V, and an electrophoresis time of approximately one hour.  

### 2.11. Observations on the Ultrastructure of Fish Muscle Fibres

The microstructural changes in fish tissues were compared and assessed by means of Masson’s trichrome staining and transmission electron microscopy (TEM). The transmission electron microscopy (TEM) method was performed in accordance with the procedure outlined by Li et al. [22], with minor adaptations. The samples were then cut into slices measuring 1 cm × 0.5 cm × 0.2 cm, and thoroughly rinsed with distilled water. They were then fixed in 2.5% solution of glutaraldehyde at a temperature of 4 °C for one hour. The samples were subjected to a series of rigorous procedures, fixed in osmium acid solution, treated with gradient dehydration in ethanol solution, treated sequentially with a mixture of acetone and resin (1:0, 3:1, 1:1, 1:3), and treated overnight with pure resin; the osmotically treated samples were heated with gradient heating in the moulds (35 °C, −60 °C, −80 °C), and finally, the embedded samples were obtained, and the samples were sliced, stained, and dried for observation on the microscope. The method of Masson’s trichrome staining [14] was slightly modified. The sections after dewaxing and water washing were stained with Weigert iron haematoxylin, rinsed with running water, and stained with acidic magenta solution; this was followed by treatment with aqueous phosphoplatinic acid, pouring off the upper solution, staining with aniline blue staining solution, rinsing with pure water, dividing with 1% glacial acetic acid in water, rinsing with pure water, dehydrating quickly with 95% ethanol, and then sealing with benzene anhydrous alcohol, xylene clear, and neutral gum for microscopic observation

### 2.12. Statistical Analysis

The analysis was performed using SPSS 26, with significance levels determined by Duncan’s test. Statistically speaking, a *p*-value less than 0.05 was deemed to be significant. The generation of graphs was facilitated by utilising the software programme Origin 2021.

## 3. Results and Discussion

### 3.1. Effect of Sodium Hypochlorite Oxidation on Tissue Protease Activity of Tilapia Fillets

#### 3.1.1. Tissue Protease B

**Figure 1:**
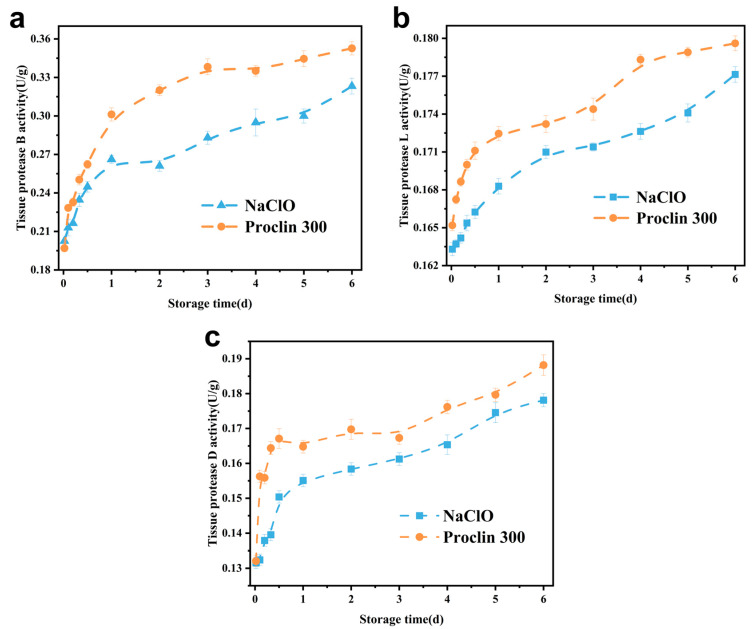
Effect of sodium hypochlorite oxidation on tissue protease activity of frozen tilapia fillets. (**a**): histatinase B; (**b**): histatinase L; (**c**): histatinase D.

The alterations in the histatinase protease B activity of tilapia fillets throughout the course of cold preservation are illustrated in Figure 1a. It can be seen that both groups showed an upward trend in histatinase B activity, which was attributed to the fact that during the postmortem stiffening of the fish, the pH was reduced, and the acidic histatinase B in the lysosomes was released and came into contact with the substrate, which in turn activated the histatinase B and elevated its enzyme activity [23]. A marked decline in histatinase B activity was observed in the sodium hypochlorite-treated group throughout the storage period when contrasted with the control group, suggesting that sodium hypochlorite treatment is capable of inhibiting histatinase B activity. It is evident that histatinase enzyme B is intimately associated with protein degradation. Therefore, the attenuation of its activity may impede protein degradation, thereby contributing to the preservation of freshness. Lan et al. [24] found that chlorine dioxide sterilisation treatment could inhibit the activity of histatinase enzyme B in rhubarb fish. This finding is congruent with the results presented in the current paper.

#### 3.1.2. Tissue Protease L

The changes in the histatinasease L activity of tilapia fillets during refrigeration are shown in Figure 1b. It can be seen that histatinasease L activity showed an increasing trend at 2–3 d, that the rise was slow, probably due to the fact that histatinasease L and histatinasease B partially reacted with the same substrate, and that the elevation of histatinase B activity partially inhibited it; in the middle and late stages of refrigeration, the enzyme activity rose faster, which was due to the fact that, accompanied by the gradual decrease in the pH of the muscle tissues, lysosomes ruptured, and a large amount of histatinasease L was released [21]. In the course of the cold storage interval, a lower tissue protease L activity was observed in the group that had been treated with sodium hypochlorite in comparison to the control group. This finding suggests that the oxidation of sodium hypochlorite has the capacity to inhibit the activity of tilapia tissue protease L.

#### 3.1.3. Tissue Protease D

The changes in the histatinasease D activity of tilapia samples stored at low temperatures are demonstrated in Figure 1c. It is evident that histatinase D activity exhibited an overall increasing trend during the process of cold storage, and the group that had undergone sodium hypochlorite treatment demonstrated a reduced level of histatinase D activity in comparison to the control group. This finding suggests that sodium hypochlorite administration exerts an inhibitor effect on histatinase D activity. This may be due to the fact that the oxidising effect of sodium hypochlorite alters the spatial structure of histatinase D, causing protein folding and hiding part of the active site of the protease, which leads to a decrease in its activity [25].

### 3.2. Effect of Sodium Hypochlorite Oxidation on the Textural Properties of Tilapia Fillets

Various textural parameters in fish meat are important indicators of the fish meat’s quality, and the main cause of changes in these parameters is the degradation of endogenous enzymes and chemical reactions in the meat. These changes are mainly manifested in alterations of the meat’s elasticity and softening [26]. As can be seen from Table 1, the elasticity, cohesion, hardness, adhesion and textural parameters of fillets from fish treated with sodium hypochlorite were higher compared to the control group on the same day. This may be explained by the action of sodium hypochlorite, which oxidises MPs, denatures proteins on the fish surface, and causes water loss and structural changes, leading to cross-linking aggregation and elevated textural parameters. After 3 d of refrigeration, the hardness, cohesion, adhesion and chewability decreased significantly, but elasticity remained stable, whereas after refrigeration up to 6 d, all textural parameters decreased significantly, which may be attributed to the degradation of fish proteins by endogenous enzymes, the weakening of intercellular bonding between muscle cells, and the disintegration of the muscle fibre structure [27], which led to the decrease in hardness, cohesion, adhesion, elasticity, and chewability, and caused the loss of fish juices and a reduction in texture. After 0–4 d of refrigeration, the hardness, adhesiveness and masticability exhibited by the sodium hypochlorite-treated specimens were considerably lower than those of the controls. This finding suggests that sodium hypochlorite may effectively retard the textural deterioration of fish by inhibiting endogenous protease activity and decreasing the rate of juice loss [28]. After 5 d of refrigeration, the differences in hardness, chewiness and elasticity between the two groups were reduced, indicating that although the oxidative sterilisation treatment with sodium hypochlorite could delay the process of protein degradation, the differences between the two groups became smaller due the continuous degradation of muscle proteins. Thus, although Proclin 300 inhibited the proliferation and reproductive capacity of microorganisms, the endogenous protease activity in the tissues remained elevated. This, in turn, resulted in a substantial decline in muscle hardness. Conversely, the sodium hypochlorite-treated group exhibited a superior capacity to preserve the quality of the fish meat morphology.

### 3.3. Effect of Sodium Hypochlorite Oxidation on Quality Characteristics of Tilapia Fillets

#### 3.3.1. Colour and Lustre

**Figure 2:**
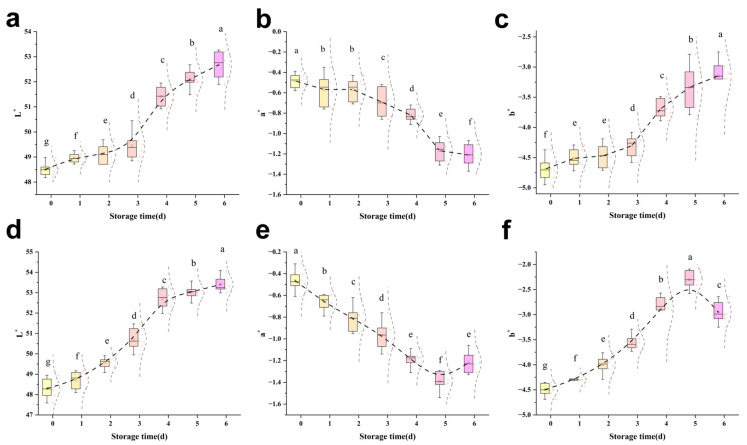
Effect of sodium hypochlorite oxidation on the colour of frozen tilapia fillets. (**a**–**c**) are sodium hypochlorite-treated groups, and (**d**–**f**) Proclin300 controls. The different letters indicate significant differences (*p* < 0.05).

Colour plays an important role in evaluating meat quality, and its stability is affected by the interrelationship between divalent iron and myoglobin [29]. As shown in Figure 2, the L* value of the sodium hypochlorite treatment group was higher than that of the control group on the same day, while the a* value was lower and the b* value was higher. This is because sodium hypochlorite oxidised oxygenated myoglobin, causing the iron ions in myoglobin to be oxidised from the ferrous state (Fe^2+^) to the ferric state (Fe^3+^). The loss of an electron by the iron ions resulted in a change in their chemical properties, transforming them into ferric myoglobin, which led to a decrease in the meat redness value. This bleaching effect of sodium hypochlorite has been demonstrated to increase the whiteness values of fish. Porter and Jänicke [25] found increased whiteness values in grass carp soaked in 300 mg/L of sodium hypochlorite for 5 min; the sodium hypochlorite solution itself was a yellowish-green colour, which may remain on the surface of the fish during the sterilisation rinsing process, resulting in a high yellowing value of the fish. During refrigeration, an increasing trend in L* values was observed in both groups of fish, which was attributed to the denaturation of myosin, the substitution of ferrous haemoglobin, alterations in the water retention capacity of the flesh, and an increase in free water on the flesh surface, which enhanced the reflected light and resulted in the disruption of the chlorophyll ring in the structure of pigmented proteins, as well as the occurrence of protein polymerisation [30]. Among other things, a* values are affected by the instability of oxygenated myoglobin in muscle, which may continue to react with oxygen to produce high iron myoglobin. As the duration of cold storage increases, the instability increases with it and the production of high iron myoglobin from oxygenated myoglobin accelerates, leading to a decrease in the a* value [31].

#### 3.3.2. pH

**Figure 3:**
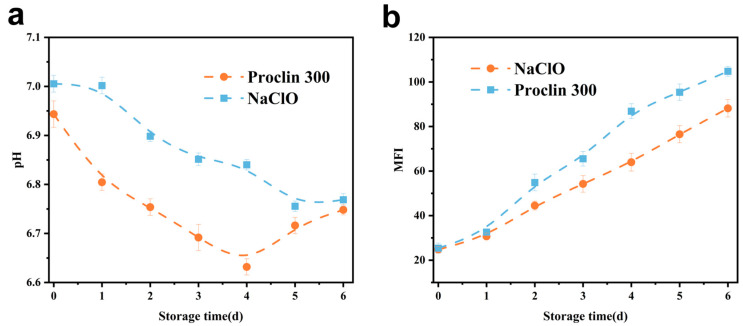
(**a**): Effect of sodium hypochlorite oxidation on pH of refrigerated tilapia fillets; (**b**): Effect of sodium hypochlorite oxidation on MFI of refrigerated tilapia fillets.

The pH change exhibited by fish meat is intimately associated with the physiological environment of its muscle tissues [23], and the detection of tilapia meat pH can be used as one of the reference standards for assessing the degree of freshness. As demonstrated in Figure 3a, the pH exhibited a decline followed by an uptick, a phenomenon attributed to the reduction in muscle pH resulting from the production of acidic by-products, including lactic acid, through the anaerobic fermentation of glycogen in vivo during the initial phase of storage. This was succeeded by an increase in the pH of fish tissues in the medial and late stages of storage, attributable to the process of protein decomposition by enzymes produced within the organism, yielding nitrogenous alkaline substances [32]. The control samples showed an inflexion point in pH after 4 d, and the sodium hypochlorite-treated group showed an inflexion point on the 5th d, indicating that the sodium hypochlorite-treated group had a slow pH and the freshness of the fish fillets was better maintained; this may be due to the fact that the oxidation of sodium hypochlorite reduces the vitality of the endogenous enzymes and diminishes their capacity to degrade the proteins, and that nitrogen-containing alkalines are slow to be stored and slow to rise in pH [33]. Overall, the sodium hypochlorite-treated group better maintained the pH in fish throughout the cold storage period.

#### 3.3.3. MFI

The phenomenon of myofibril fragmentation refers to the formation of smaller fragments in myofibrils as a result of the gradual deterioration of myosin and concomitant actins, which leads to myofibrils breaking near the Z line [34]. As illustrated in Figure 3b, the MFI of the various treatment groups exhibited an upward trend, consistent with the findings reported by Xiong et al. [35] for Siniperca chuatsi and by Lan et al. [36] for perch. However, the magnitude of these changes was minimal, attributed to the slower rate of bacterial reduction, which resulted in a reduced disruption of the myofibrillar fibres’ internal structure. The MFI of the sodium hypochlorite-treated group was lower compared to the control group throughout the observed storage period, with minimal alterations observed in the initial phase. This finding suggests that the oxidising effect of sodium hypochlorite was insufficient to induce substantial damage to myofibrils and their skeleton [37], but on the contrary, it inhibited the degradation of MP, which helped to preserve the structural stability exhibited by myofibrils. Moreover, the inhibition of MP degradation may be attributed to the inhibitory effect of sodium hypochlorite on the endogenous protease activity, thereby reducing the extent of protein degradation.

### 3.4. Effect of Sodium Hypochlorite Oxidation on the Protein Structure of Tilapia Fillets

#### 3.4.1. SDS-PAGE

**Figure 4:**
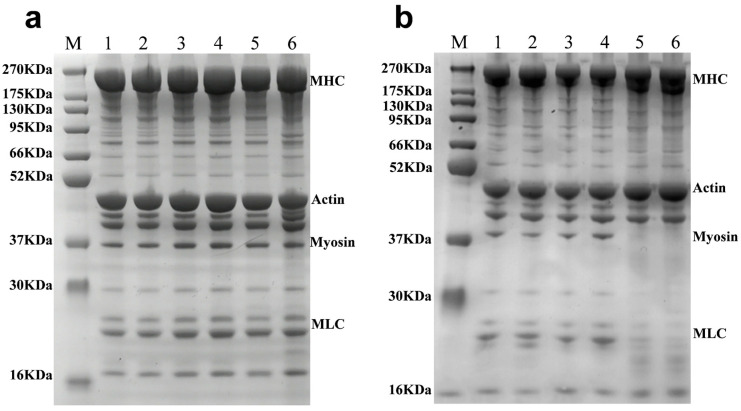
Effect of sodium hypochlorite oxidation on SDS-PAGE of frozen tilapia fillet proteins. (**a**) sodium hypochlorite-treated group; (**b**) Proclin 300 control group; M, 1, 2, 3, 4, 5, and 6 denote marker, refrigerated for 1, 2, 3, 4, 5, and 6 d.

Changes in the MP of sodium hypochlorite-treated tilapia fillets during refrigerated storage have been demonstrated in Figure 4a. From 0 to 4 d, the myosin heavy chain strength gradually weakened, there was no significant difference between actin and pro-myosin, and the degradation of myosin light chain occurred. The protein degradation of the Proclin 300 control group is shown in Figure 4b. On the 5th–6th d of refrigeration, MP underwent a significant degradation, with a significant reduction in myosin heavy chain strength, and the complete degradation of pro-myosin and myosin light chain. These findings are consistent with the results reported by Xu et al. [38], which indicated a gradual decrease in the relative content of actin (43 kDa molecular weight) with an increase in storage time. In the sodium hypochlorite-treated group (Figure 4a) at 6 d of cold storage, the intensity of myosin heavy chain was reduced in the sodium hypochlorite-treated group, and new bands were degraded from 16 to 30 kDA and 37 to 52 kDA, indicating that the protein produces significant degradation [39]. Yan et al. [40] reported that the thinning of MHC and myosin binding is associated with MP degradation, indicating that fish protein gradually degrades into peptides or other small molecules. Throughout the freezing storage process, compared to the control group, the protein bands of MHC, bands I and II, actin, myosin, and MLC in the muscle filament proteins of tilapia showed a gradual decrease in intensity and the narrowing of the bands, indicating that the degradation of MP in the sodium hypochlorite oxidation treatment group was significantly lower than that in the control group, suggesting that sodium hypochlorite washing has an inhibitory effect on protein denaturation and degradation [41], inhibiting histatinase activity and reducing protein degradation [42], consistent with the conclusions in Section 3.1.

#### 3.4.2. SEM

**Figure 5:**
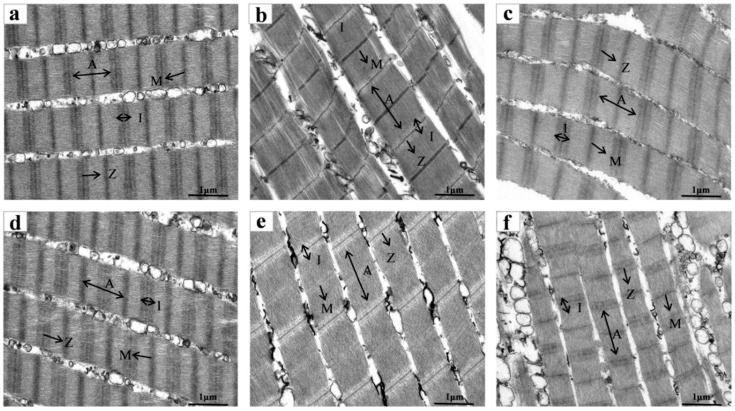
Effect of sodium hypochlorite oxidation on the ultrastructure of refrigerated tilapia fillets (10,000×). (**a**–**c**) sodium hypochlorite treatment for 0, 3, and 6 d of refrigeration; (**d**–**f**) control treatment for 0, 3, and 6 d of refrigeration. Letters represent the characteristic structure of proteins.

The effect of sodium hypochlorite oxidation on the ultrastructure of the myofibers of tilapia fillets is shown in Figure 5. On the day of treatment, both the sodium hypochlorite-treated group and the Proclin 300 control group had clearly characterised H-regions, I-bands, M-lines, A-bands and Z-lines, and both Z-lines and M-lines were aligned neatly, with myogenic fibres tightly connected. As can be seen from the sarcomeres diagrams of Figure 5a–c, the sarcomeres of the fish did not change significantly during cold storage, and the M-band was still clear, and began to blur at 6 d of storage in the Z-disc, which may be due to the degradation of the Z-disc. Since the Z-line plays a role in connecting adjacent myonodal segments, the disruption of the structure of the Z-line resulted in the disorganisation of the myofibrils of the tilapia fillet muscle fibre, which indicated that the myofibrillar proteins of the treatment group had been degraded by 6 d, and this was in agreement with the SDS- PAGE results (Figure 4). As can be seen from the ultrastructural analysis of tilapia fillets in Figure 5d–f, at d 3, the M-line became blurred; at d 6, the Z-line of the control group became blurred, and the M-line also appeared to be weakened and distorted in its arrangement. In addition, the structural proteins of the myofibrils degraded a great deal at the late stage of refrigeration, which led to the disintegration of the M-line and Z-disc, the blurring of the light and dark regions I-band and A-band, and the fragmentation of myofibrils with the loss of integrity in the muscle cells.

**Figure 6:**
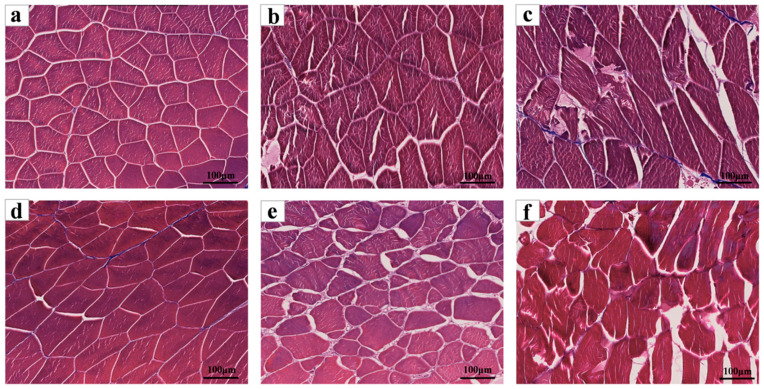
Morphological changes in muscle fibres of chilled tilapia fillets under different treatment conditions after Masson’s trichrome staining (100×). (**a**–**c**) sodium hypochlorite treatment chilled for 0, 3, and 6 days, respectively; (**d**–**f**) the control group treated and chilled for 0, 3, and 6 days, respectively.

The results of Masson’s trichrome staining are shown in Figure 6. The muscle fibre structure on the back of tilapia fillets on the day of treatment was complete and tightly arranged, with smaller and uniform gaps between fibres and clear outlines. Through observation, on the 3rd day of cold storage, the muscle fibre structure was more complete, the muscle fibre structure was relatively intact, with increased space between muscle fibres, and the connective tissue was gradually degrading and presenting a reticular porous structure. Furthermore, the integrity of muscle fibres subjected to sodium hypochlorite treatment was maintained to a greater extent than those in the control group. Following a six-day refrigeration period, a significant alteration in the ultrastructure of the tilapia fillets in the control group was observed. This alteration manifested as a fracturing and twisting of the local fibre structure, an expansion of the gap between muscle segments, and a general loosening of the structure. Muscle fibre degradation is severe, as reflected in the space between muscle fibres, whose shape became irregular, with some of them broken. In contrast, the sodium hypochlorite-treated group exhibited a larger cellular gap on the third day of refrigeration, yet its overall structure remained more intact, with muscle fibres still arranged in parallel. The enhanced integrity of muscle cells observed in the sodium hypochlorite-treated group can be ascribed to the suppression of myogenic fibre and connective tissue degradation by endogenous proteases in fish [43]. The morphological results of muscle knots indicated that sodium hypochlorite treatment inhibited the deterioration process of fish meat.

### 3.5. Correlation Analysis Between Tissue Protease Activity and Various Physicochemical Indices of Fish Flesh

**Figure 7:**
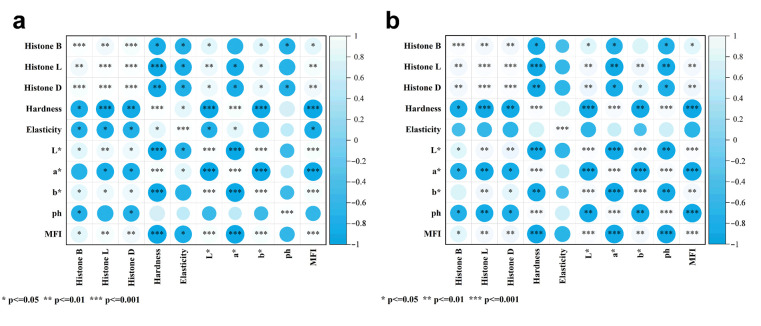
Correlation analysis of enzyme activity with various physicochemical indices. (**a**) Sodium hypochlorite-treated group, (**b**) Proclin 300 control group.

Pearson’s correlation analysis was conducted between histatinase activity and the fish hardness, MFI and pH of tilapia fillets throughout the period of cold storage. The results are presented in Figure 7 for the sodium hypochlorite-treated groups, as follows: Histatinase L and D exhibited a correlation with fish hardness and pH that was deemed to be highly significant (*p* < 0.01), and histatinase B demonstrated a correlation with hardness and MFI that was considered to be significant (*p* < 0.05). In the control group, significantly negative correlations were identified between histatinase B and hardness (*p* < 0.05), as well as between L and D (*p* < 0.01). Additionally, MFI exhibited a marked positive association with histatinase B (*p* < 0.05) and a highly significant positive association with L and D (*p* < 0.05). As demonstrated in Figure 1, a statistically significant negative correlation was observed between pH and histatinase B (*p* < 0.01), and a significant negative correlation was identified between pH and L and D (*p* < 0.05). Furthermore, MFI was found to be significantly positively correlated with hardness and pH, and highly significantly negatively correlation with L and D (*p* < 0.05). In the context of the prevailing experimental conditions, it was determined that histatinase B, L and D constituted the pivotal endogenous enzymes influencing the quality alterations undergone by tilapia throughout the cold storage process following bacterial reduction.

## 4. Conclusions

This study investigated the effect of sodium hypochlorite disinfectant on the quality of tilapia fillets during storage. The results showed that during refrigeration, the activity of tissue proteases B, L, and D in the sodium hypochlorite-treated group was lower than that in the control group. It can be concluded that the decrease in enzyme activity was caused by the oxidative action of sodium hypochlorite. This finding suggests that the reduction in enzyme activity was attributable to the oxidising nature of sodium hypochlorite. In comparison with specimens in the control group, those treated with sodium hypochlorite exhibited the diminished degradation of myosin heavy chains, pro-myosin, and muscle light chains. Additionally, the deterioration of texture and colour was reduced, and the structural integrity of fish muscle cells was enhanced. The index of myofibril fragmentation was also reduced in the treated specimens. Correlation analysis revealed a negative correlation between histatinase B, L and D and the hardness of fish meat, and a positive correlation with the MFI, which indicated that histatinases play a significant role in the quality change in tilapia fillets in the process of refrigeration. In addition, sodium hypochlorite reduces the degradation of fish protein by inhibiting histatinase activity, and reduces the structural integrity of the fish muscle fibres by reducing the rate of deterioration in the tilapia fish fillets during storage. This experiment provides data that supports the improvement in the storage quality of tilapia, and provides a new perspective for improving the storage quality of tilapia.

## Figures and Tables

**Table 1:** Effect of sodium hypochlorite oxidation on the texture of frozen tilapia fillets. A: Sodium hypochlorite-treated group, B: Proclin300 control group.

Storage Time (d)		0	1	2	3	4	5	6
Hardness/Pa	A	342.83 ± 23.01 ^a^	316.96 ± 16.88 ^b^	288.78 ± 12.20 ^c^	265.25 ± 11.23 ^d^	237.56 ± 13.41 ^e^	208.24 ± 20.23 ^f^	189.83 ± 16.11 ^g^
	B	338.83 ± 17.05 ^a^	298.85 ± 14.25 ^b^	269.23 ± 25.36 ^c^	229.36 ± 16.85 ^d^	207.9 ± 17.45 ^e^	182.52 ± 15.25 ^f^	171.56 ± 18.10 ^g^
Cohesion	A	0.50 ± 0.01 ^a^	0.46 ± 0.05 ^b^	0.42 ± 0.02 ^c^	0.42 ± 0.04 ^c^	0.37 ± 0.07 ^d^	0.35 ± 0.05 ^e^	0.35 ± 0.04 ^e^
	B	0.49 ± 0.01 ^a^	0.44 ± 0.03 ^b^	0.40 ± 0.01 ^c^	0.39 ± 0.03 ^d^	0.36 ± 0.07 ^e^	0.35 ± 0.02 ^f^	0.36 ± 0.08 ^e^
Elasticity/mm	A	3.54 ± 0.08 ^a^	3.53 ± 0.02 ^b^	3.53 ± 0.09 ^b^	3.51 ± 0.09 ^d^	3.52 ± 0.07 ^c^	3.51 ± 0.02 ^d^	3.48 ± 0.37 ^e^
	B	3.53 ± 0.02 ^a^	3.52 ± 0.07 ^b^	3.52 ± 0.05 ^b^	3.51 ± 0.01 ^c^	3.50 ± 0.06 ^d^	3.51 ± 0.09 ^c^	3.42 ± 0.56 ^e^
Adhesion	A	132.56 ± 2.47 ^a^	120.10 ± 1.89 ^b^	110.29 ± 12.49 ^c^	105.75 ± 2.90 ^d^	88.9 ± 11.85 ^e^	60.45 ± 12.25 ^g^	63.4 ± 9.6 ^f^
	B	132.23 ± 2.28 ^a^	119.23 ± 2.64 ^b^	101.26 ± 2.56 ^c^	96.55 ± 1.87 ^d^	62.85 ± 13.1 ^e^	46.25 ± 8.9 ^f^	44.3 ± 7.7 ^g^
Chewability/mJ	A	4.59 ± 0.13 ^a^	3.96 ± 0.43 ^b^	3.58 ± 0.25 ^c^	3.02 ± 0.28 ^d^	2.87 ± 0.51 ^e^	2.56 ± 0.43 ^f^	2.23 ± 0.56 ^g^
	B	4.58 ± 0.37 ^a^	3.25 ± 0.29 ^b^	2.75 ± 0.14 ^c^	2.57 ± 0.11 ^d^	2.22 ± 0.88 ^e^	2.15 ± 0.12 ^f^	1.68 ± 0.72 ^g^

Note: Data are expressed as mean ± standard deviation (n = 6); a letter superscripted to a number in the same row indicates a significant difference between groups (*p* < 0.05).

## Data Availability

The original contributions presented in this study are included in the article. Further inquiries can be directed to the corresponding authors.
